# Comparison of four MR carotid surface coils at 3T

**DOI:** 10.1371/journal.pone.0213107

**Published:** 2019-03-04

**Authors:** Qinwei Zhang, Bram F. Coolen, Sandra van den Berg, Gyula Kotek, Debra S. Rivera, Dennis W. J. Klomp, Gustav J. Strijkers, Aart J. Nederveen

**Affiliations:** 1 Amsterdam UMC, University of Amsterdam, Radiology and Nuclear Medicine, Amsterdam, the Netherlands; 2 Amsterdam UMC, University of Amsterdam, Biomedical Engineering and Physics, Amsterdam, the Netherlands; 3 Department of Radiology & Nuclear Medicine, Erasmus MC, University Medical Center, Rotterdam, The Netherlands; 4 Spinoza Centre for Neuroimaging Amsterdam, Amsterdam, The Netherlands; 5 Department of Radiology, UMC Utrecht, the Netherlands; Henry Ford Health System, UNITED STATES

## Abstract

**Background:**

The quality of carotid wall MRI can benefit substantially from a dedicated RF coil that is tailored towards the human neck geometry and optimized for image signal-to-noise ratio (SNR), parallel imaging performance and RF penetration depth and coverage. In last decades, several of such dedicated carotid coils were introduced. However, a comparison of the more successful designs is still lacking.

**Objective:**

To perform a head-to-head comparison over four dedicated MR carotid surface coils with 4, 6, 8 and 30 coil elements, respectively.

**Material and methods:**

Ten volunteers were scanned on a 3T scanner. For each subject, multiple black-blood carotid vessel wall images were measured using the four coils with different parallel imaging settings. The performance of the coils was evaluated and compared in terms of image coverage, penetration depth and noise correlations between elements. Vessel wall of a common carotid section was delineated manually. Subsequently, images were assessed based on vessel wall morphology and image quality parameters. The morphological parameters consisted of the vessel wall area, thickness, and normalized wall index (wall area/total vessel area). Image quality parameters consisted of vessel wall SNR, wall-lumen contrast-to-noise ratio (CNR), the vessel g-factor, and CNR_index_ ((wall–lumen signal) / (wall+lumen signal)). Repeated measures analysis of variance (rmANOVA) was applied for each parameter for the averaged 10 slices for all volunteers to assess effect of coil and SENSE factor. If the rmANOVA was significant, post-hoc comparisons were conducted.

**Results:**

No significant coil effect were found for vessel wall morphological parameters. SENSE acceleration affected some morphological parameters for 6- and 8-channel coils, but had no effect on the 30-channel coil. The 30-channel coil achieved high acceleration factors (10x) with significantly lower vessel g-factor values (*p*s ≤ 0.01), but lower vessel wall SNR and CNR values (*p*s ≤ 0.01).

**Conclusion:**

All four coils were capable of high-quality carotid MRI. The 30-channel coil is recommended when rapid image acquisition acceleration is required for 3D measurements, whereas 6- and 8-channel coils demonstrated the highest SNR performance.

## Introduction

Carotid MRI is increasingly used in clinical research as a non-invasive tool for studying atherosclerotic disease [1–6]. High-resolution images with typical isotropic voxel dimensions of 0.7 mm or smaller are required to reveal the morphology of the carotid vessel wall plaque components [7]. At the same time, adequate image signal-to-noise ratio (SNR), sufficient coverage along carotid vessels, short imaging duration, and robust reproducible imaging protocols are desired to make carotid plaque MRI feasible for routine clinical use. To meet these requirements, many dedicated carotid surface coils were developed by several companies and researchers over the past decades for improving image quality and scan efficiency above the standard vendor-provided coils which are often not specifically optimized for the neck anatomy [8]. These advances have also proven crucial for the recent introduction of quantitative carotid imaging techniques, such as vessel wall tissue parameter mapping [9–12] and carotid flow and wall shear stress imaging [13–15]. Such techniques rely even more on high SNR and fast parallel imaging to prevent lengthy protocols and guarantee accurate parameter estimation. Moreover, achieving higher SNR and parallel imaging acceleration also increases the reproducibility of carotid imaging outcome measures for large cohort and multi-center studies [16–18].

In 1996, Hayes *et al*. proposed the use of a 4-channel flexible phase array coil with a channel element size of 6.4 cm × 6.4 cm. This coil was easy to position around the neck and was well tolerated by patients [19]. Liffers *et al*. later optimized the position and the size of the 4-channel coil elements to increase the SNR performance [20]. In 2007, *Ouhlous et al*. demonstrated that a quadrature coil, consisting of a circular single-loop sandwiched by a butterfly loop, outperformed the 4-channel design in terms of SNR at 3 cm depth [21]. Afterwards, Balu *et al*. proposed an 8-channel coil with a total size of 12.8 cm × 10.3 cm for each side. This coil proved to be superior to the 4-channel coil showing higher SNR at typical carotid depth, improved vessel visualization and a larger coverage [22]. More recently, a high density 30-channel array surface coil for 7T carotid imaging was proposed by Koning *et al*. with a total dimension of 10 cm × 15 cm for each side and a coil element size of 1.5 cm × 3.5 cm. With this coil, high parallel acceleration factors of 4 in two directions could be obtained [23].

Despite the use of the abovementioned carotid coils in recent studies, a head-to-head assessment of their performance was never done. In this study, we therefore performed a quantitative comparison of the performance of four different carotid surface coils, which we have available in our institute. These are a 4-channel coil, a 6-channel quadrature butterfly coil, an 8-channel coil and a 30-channel coil. For all coils, both SNR and parallel imaging performances were evaluated *in vivo* on 10 volunteers. Additionally, carotid vessel wall morphological parameters were compared for the different coil setups.

## Materials and methods

### Coil descriptions

**Fig 1:**
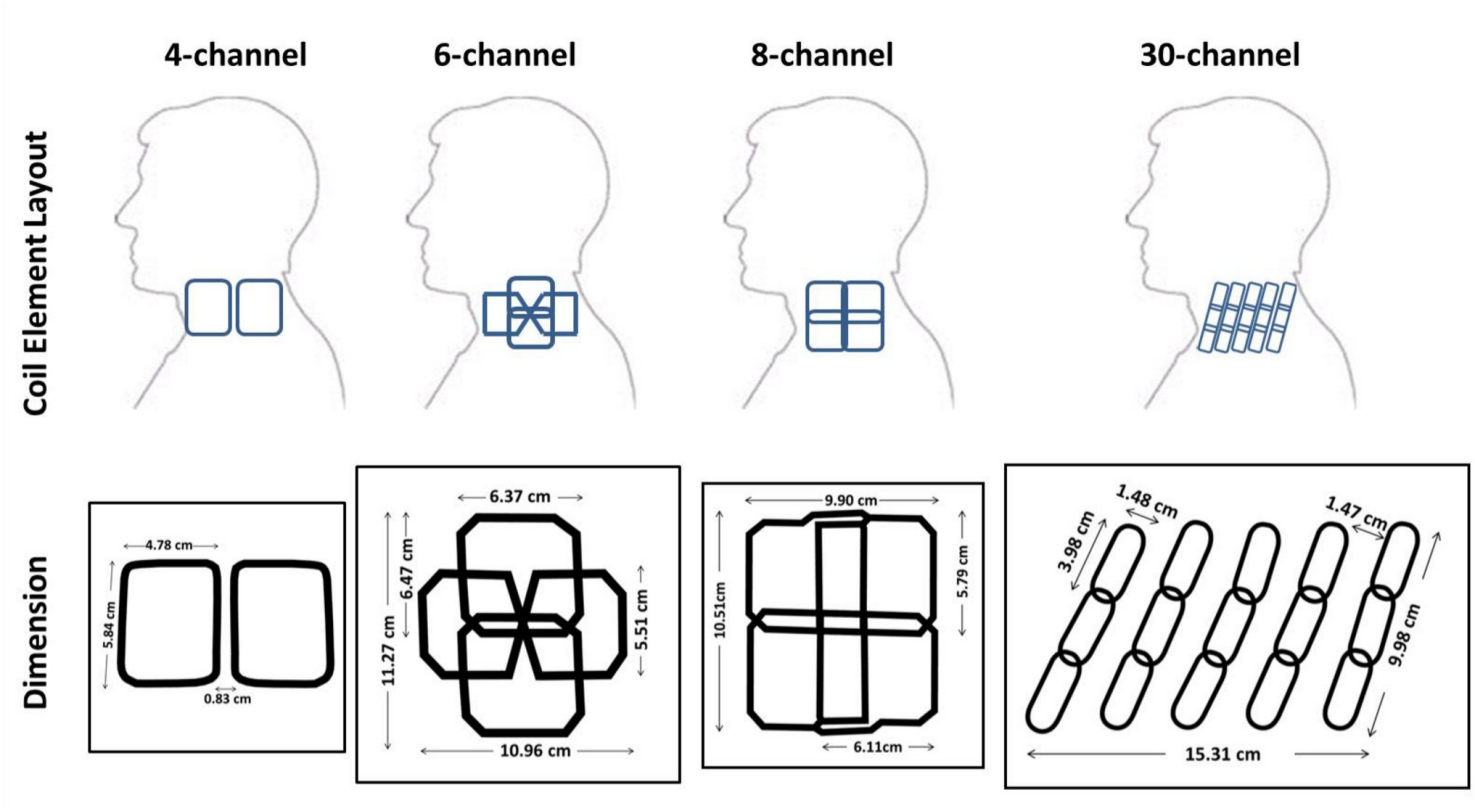
Unilateral (right side) layouts and dimensions of the four investigated coils. All coils consist of two identical parts for left and right sides.

Four dedicated carotid MRI phased-array surface coils with 4, 6, 8, and 30 receiving channels from 3 vendors were investigated. Because we only had a unilateral setup available for the 30-channel coil (i.e. 15 coil elements), experiments with the other coils were also performed unilaterally using only coil elements on the right side of the neck (i.e. 2, 3 or 4 elements). To avoid confusion when comparing the number of coil elements, throughout the manuscript we keep referring to the number of elements present in the bilateral setup. Coil element layouts and dimensions are illustrated in Fig 1. Coil dimensions were measured by means of a computed tomography (CT) scan.

The 4-channel coil (Machnet B.V., Roden, The Netherlands) has a similar design as described previously by Hayes *et al*. [19]. Its coil elements have a size of 5.48 cm × 4.78 cm separated by 0.83 cm in the anterior-posterior (AP) direction to reduce the noise correlation. The 6-channel coil is a prototype built in the Erasmus Medical Center (Rotterdam, the Netherlands) in collaboration with Machnet B.V. and Flick Engineering B.V. (Winterswijk, The Netherlands) and optimized for measuring carotid vessels at a depth of 2–3 cm. Each side of the 6-channel coil consists of 2 normal single-loop coil elements (6.47 cm × 6.37 cm) slightly overlapped in the feet-head (FH) direction and a butterfly coil element (5.51 cm × 10.96 cm) placed along the AP direction. Separate preamplifiers were used for individual coil element to suppress the mutual inductance, resulting in more freedom for coil element position. The 8-channel coil (Shanghai Chenguang Medical Technologies, China) has a similar design as described earlier by Balu *et al*. [22] with a coil element size of 6.11 cm × 5.79 cm. Coil elements were overlapped to minimize both the mutual inductance as well as the signal drops between coil elements. The 30-channel coil (MR Coils B.V., the Netherlands) consists of five columns of three-element groups on each side. Coil elements have a size of 1.48 cm × 3.98 cm and overlap 0.4 cm in the FH direction to minimize signal drops in between the elements. To optimize parallel imaging ability in the AP direction, columns are separated by 1.47 cm. Preamplifier decoupling was used to minimize the mutual inductance.

### *In vivo* measurement

Ten healthy volunteers were scanned with a 3T scanner (Ingenia, Philips, Best, the Netherlands) within an 8 week-period (9 males and 1 female; age range: 21–36 y, mean ± SD = 25.6 ± 4.5 y). Amsterdam UMC (AMC) medical ethics committee approved the study (approval number: W15_373 # 16.007). Written informed consent was obtained from all volunteers.

For each coil, the same scanning protocol was performed. Between measurements with different coils, volunteers were taken off from the scanner and repositioned on the MRI table. The coils were placed by experienced radiographers. The order of the coils used for scanning was randomized over the volunteers. All the volunteers in this study were asked to keep their necks in the same position as much as possible to keep the carotid depth identical between coils. In order to visualize the position of the coils during scan planning, small oil capsules were attached to the coils.

3D transversal black blood carotid vessel wall images of the right carotid artery were obtained using the 3D Motion‐Sensitized Driven Equilibrium (MSDE) prepared Rapid Gradient Echo (3D‐MERGE) sequence [9, 24]. The following imaging parameters were used: FOV = 150 (AP) × 150 (LR: left-right) × 100 (FH) mm^3^, voxel size = 0.7 × 0.7 × 2 mm^3^, turbo field echo (TFE) factor = 60, flip angle = 6°, MSDE preparation length = 12 ms, velocity encoding (venc) = 3 cm/s, no parallel imaging, total scan time = 3 min 30 s. Corresponding noise images were obtained by repeating the sequence with RF at zero power.

To fully explore the parallel imaging (SENSE acceleration) ability of the coils, additional SENSE accelerated scans were performed by modifying the SENSE factor of the 3D‐MERGE measurement described above. In all scans, frequency, phase and slice encoding directions of the 3D‐MERGE measurement were chosen to be LR, AP and FH, respectively. Different SENSE acceleration settings for the 6-channel coil (AP-FH: 1–2), the 8-channel coil (AP-FH: 1–2; 2–1; 2–2) and the 30-channel coil (AP-FH: 1–2; 2–1; 1–3; 2–2; 2–3; 2–4; 2.5–4; 4–2.5) were used based on their coil element layouts.

#### SNR, g-factor and noise correlation calculation

SNR maps for non-accelerated scans were calculated as the ratio of the image intensity to the local noise level as

$$SNR(r)\;=\;\frac{S_{image}(r)}{1.5\;∙\;SD(n_{kernel}(r))}\;,$$ (1)

where S_image_ is the signal intensity at location **r**, n_kernel_ is a 7 × 7 × 7 local noise kernel around location **r**, SD is the standard deviation of the noise in the kernel. The SD value of the noise intensity is scaled 1.5 times to calibrate the skewness of the Rayleigh noise distribution [25]. SNR maps for accelerated scans were calculated indirectly based on the non-accelerated SNR maps and the corresponding g-factor maps, as

$${SNR}_{acc}\;=\;\frac{{SNR}_{nonacc}}{{\;g}_{R\;}∙\;\sqrt{R}}\;,$$ (2)

where SNR_nonacc_ and SNR_acc_ are the SNR of non-accelerated scan and accelerated scan, respectively, R is the SENSE acceleration factor and g_R_ is the g-factor. Unlike the SNR calculation for non-accelerated measurement, noise images for SENSE accelerated scans were not used, as they were affected by many SENSE reconstruction settings and the SENSE reference scan and consequently deemed unreliable for reflecting the true noise level. In comparison, the g-factor calculation was independent of these settings.

In this study, the g-factor map was generated automatically by the scanner software during the SENSE reconstruction as

$$g_{R}\;=\;\frac{1}{\sqrt{R}}\frac{\sqrt{H_{acc\;}{Ѱ}_{acc}H_{acc}^{*}}}{\sqrt{H_{nonacc\;}{Ѱ}_{nonacc}H_{nonacc}^{*}}}\;,$$ (3)

where *H*_*nonacc*_ and *H*_*acc*_ denote channel combination matrices of non-accelerated and accelerated scan, respectively; Ψ_*nonacc*_ and Ψ_*acc*_ denote noise covariance matrices for non-accelerated and accelerated scan, respectively [26].

Noise correlation matrices were calculated from channel-by-channel noise images as

$$\psi_{i,j}\;=\;\frac{\sum_{r}{[(n}_{i}(r)-{\overset{-}{n}}_{i})\;∙\;(n_{j}(r)-{\overset{-}{n}}_{j})]}{\sqrt{\sum_{r}{{(n}_{i}(r)-{\overset{-}{n}}_{i})}^{2}\;\cdot \;\sum_{r}{{(n}_{j}(r)-{\overset{-}{n}}_{j})}^{2}}}\;,$$ (4)

where ψ_i,j_ is the correlation between i^th^ channel and the j^th^ channel, n_i_(**r**) is the noise intensity at location **r** in i^th^ channel, ${\overset{-}{n}}_{i}$ is the mean noise intensity of i^th^ channel.

#### Image analysis

Image analysis was performed in VesselMASS (Medis, Leiden, the Netherlands). Images were interpolated to a 0.35 × 0.35 mm^2^ in-plane resolution. For every 3D image volume, the right common carotid artery vessel wall was manually delineated in 10 image slices below the bifurcation by a radiographer with 10 years of experience (S). First, vessel wall morphological parameters were assessed including vessel wall area (A_vw_), horizontal vessel wall thickness (T_h_) and vertical vessel wall thickness (T_v_). In addition, the normalized wall index (NWI) defined as A_vw_ / A_vessel_ was calculated, where A_vessel_ is the total vessel area. Second, image quality parameters were assessed, including vessel wall SNR, vessel wall-lumen contrast-to-noise ratio (CNR) and the vessel g-factor. The CNR was calculated as the difference between the vessel wall SNR and the lumen SNR. Furthermore, an extra CNR quantification parameter (CNR_index_), independent of g-factor measurement, noise measurement and image scaling factor, was calculated as

$${CNR}_{index}\;=\;\frac{S_{vw}-\;S_{lumen}}{S_{vw}+\;S_{lumen}}\;,$$ (5)

**Fig 2:**
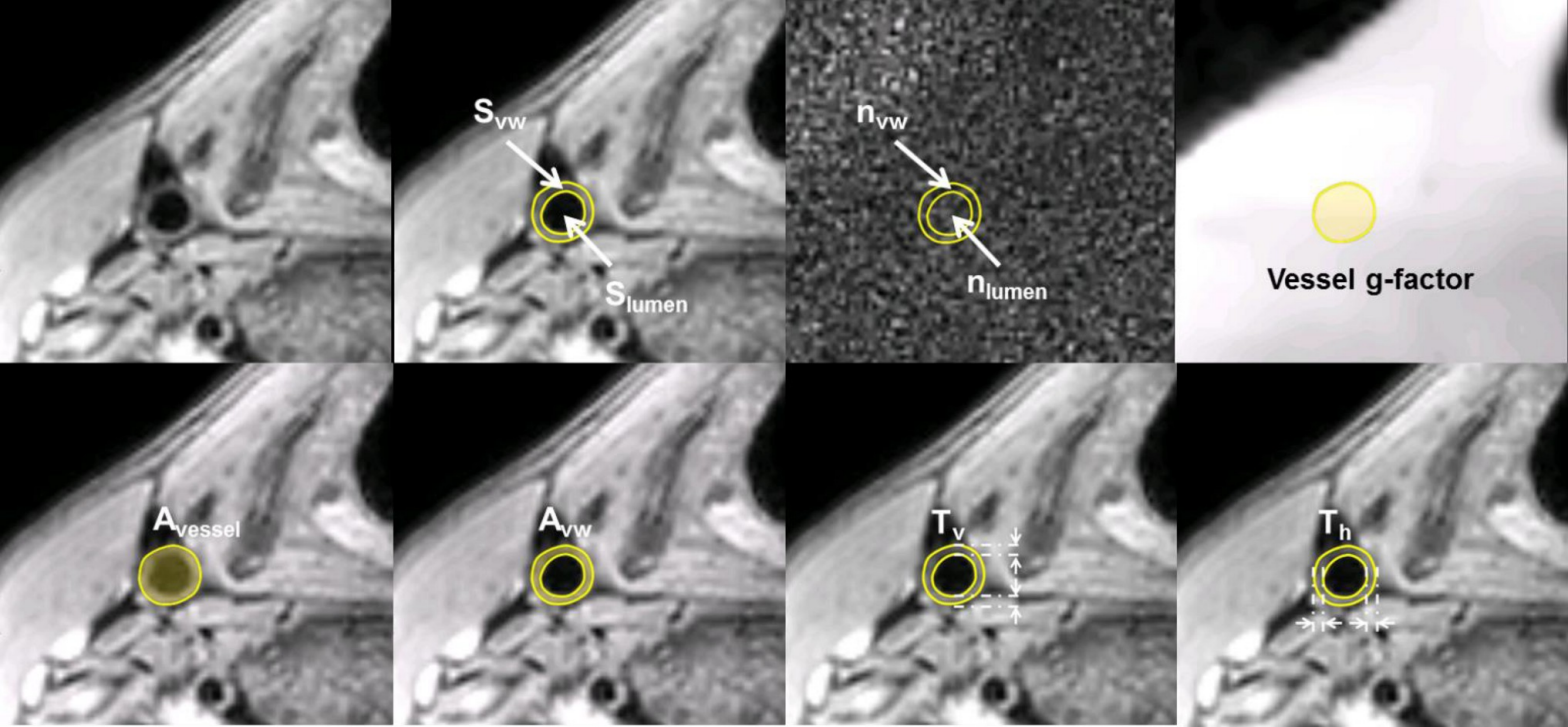
Example of vessel wall annotations (images were acquired with the 4-channel coil). Vessel wall signal intensity (S_vw_), lumen signal intensity (S_lumen_), vessel area (A_vessel_), vessel wall area (A_vw_), vertical vessel wall thickness (T_v_) and horizontal vessel wall thickness (T_h_) were measured on the on anatomical 3D‐MERGE images. Vessel wall noise level (n_vw_), lumen noise level (n_lumen_) were measured on the noise images using the S_vw_ and S_lumen_ ROIs. Vessel g-factor was measured on g-factor maps using the A_vessel_ ROI.

Image evaluation parameters are annotated on the 3D‐MERGE image, the noise map and the g-factor map as shown in Fig 2.

#### Statistical analysis

All statistical tests were performed in SPSS 22 (SPSS Inc., Chicago, IL, USA) at a p < 0.05 significance level. Repeated measures analysis of variance (rmANOVA) was applied for each parameter for the averaged 10 slices of all volunteers. In case of a significant ANOVA (multivariate F-test), post-hoc comparisons with Sidak’s correction were used to compare which pairs differed significantly. The rmANOVA was used to assess the effect of coil (without SENSE acceleration) and the effect of SENSE acceleration (within coils).

## Results

**Fig 3:**
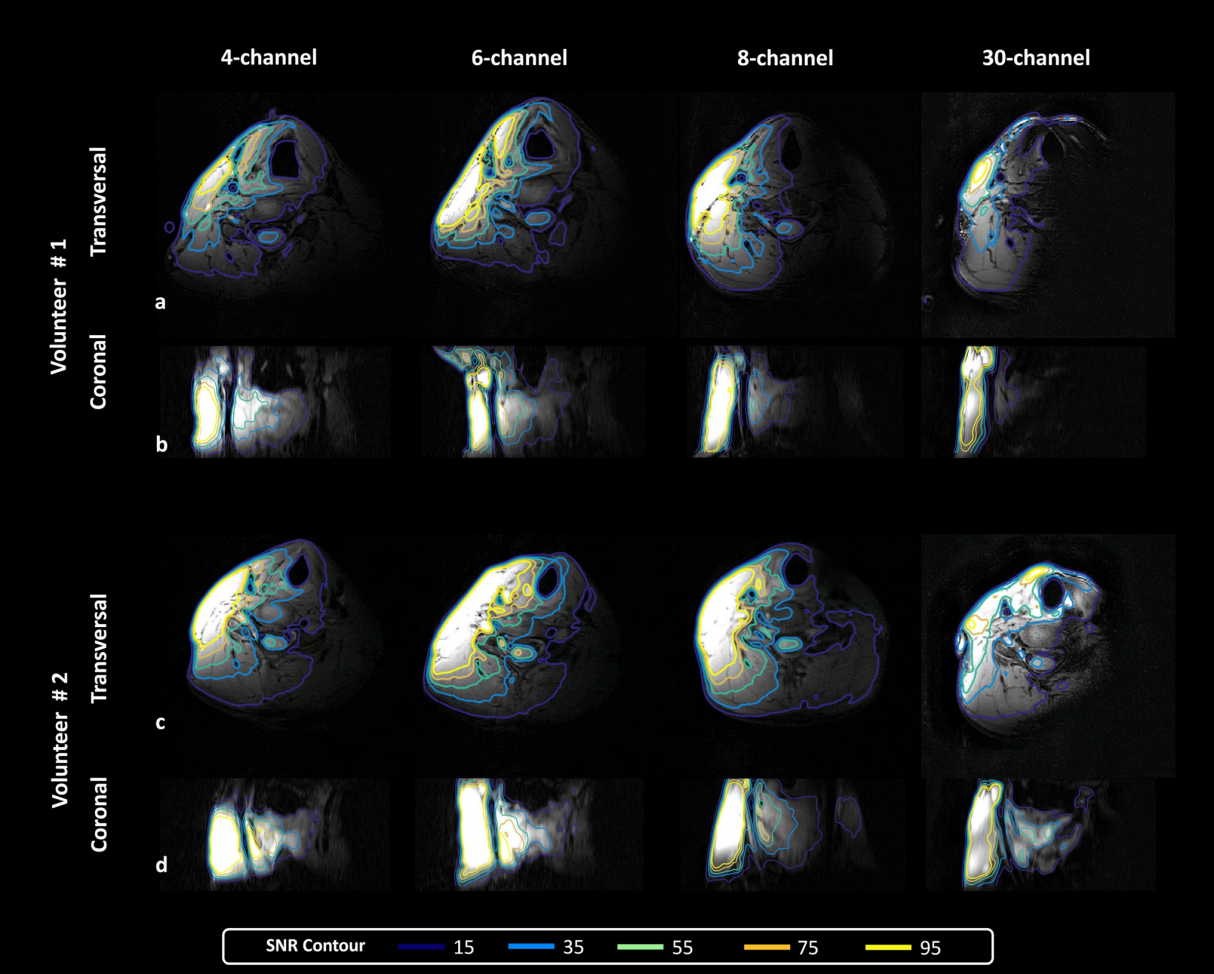
Representative transversal and coronal 3D‐MERGE images of the neck from two volunteers obtained by different coils without SENSE acceleration. Five SNR contours ranging from 15 to 95 are overlaid on the images. Here all coils were used as a unilateral setup resulting in coverage of one side of the neck.

Fig 3 shows representative *in vivo* non-accelerated 3D‐MERGE images from 2 volunteers with overlaid SNR contours. In the transversal orientation, the 4-, 6- and 8-channel coils had similar SNR contour areas with the exception for the 8-channel coil of volunteer #2, which had a much larger SNR-15 contour. In contrast, the 30-channel coil had less penetration at all SNR levels. In the coronal view, this difference was less obvious. For all coils, a minimal SNR of 15 was reached for the entire 100-mm carotid vessel wall section in the FH direction. The 4-channel coil had shorter SNR > 95 coverage in the FH direction compared with other coils. Additionally, all coils had SNR-15 contours larger than half of the FOV and achieved SNR > 75 in muscle tissue between the carotid artery and the coil, such as in the scalene muscles and the sternocleidomastoid (SCM) muscle.

**Fig 4:**
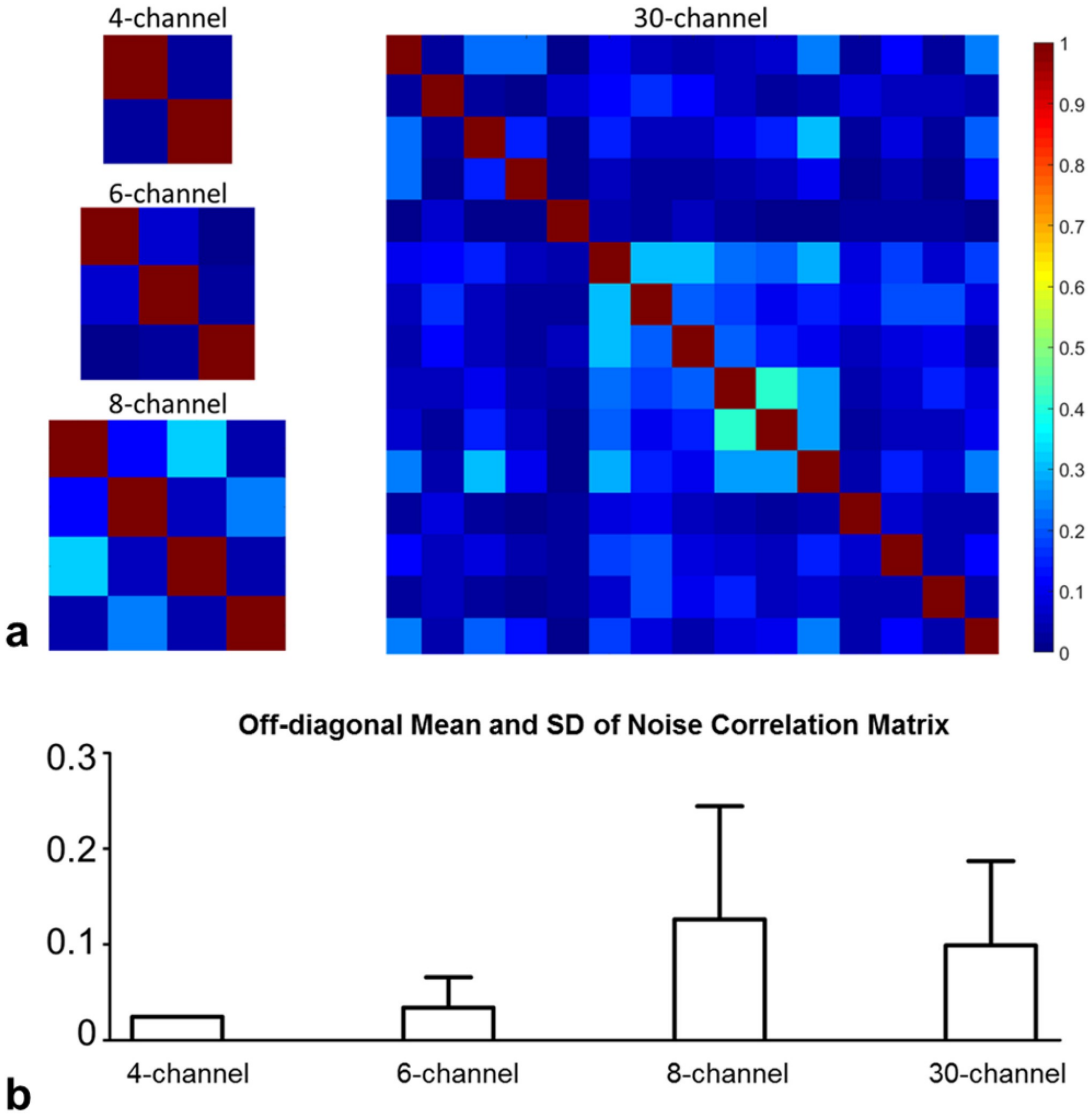
Noise correlation between different coil elements. (**a**) Noise correlation matrices for the four coils. (**b**) Bar plots for the noise correlation values in **a**. The size of the correlation matrices corresponds to the number of coil elements in the unilateral setup.

Noise correlation between coil elements is illustrated in Fig 4. The 4- and 6-channel coils demonstrated low noise correlation with mean and SD of 0.03 ± 0 and 0.03 ± 0.03, respectively. In comparison, noise correlation for the 8- and 30-channel coils were 0.13 ± 0.12 and 0.10 ± 0.09, respectively. Note that for the 30-channel coil, the noise correlation between channel 9 and channel 10 was much higher than for other channels with a value of 0.41.

**Fig 5:**
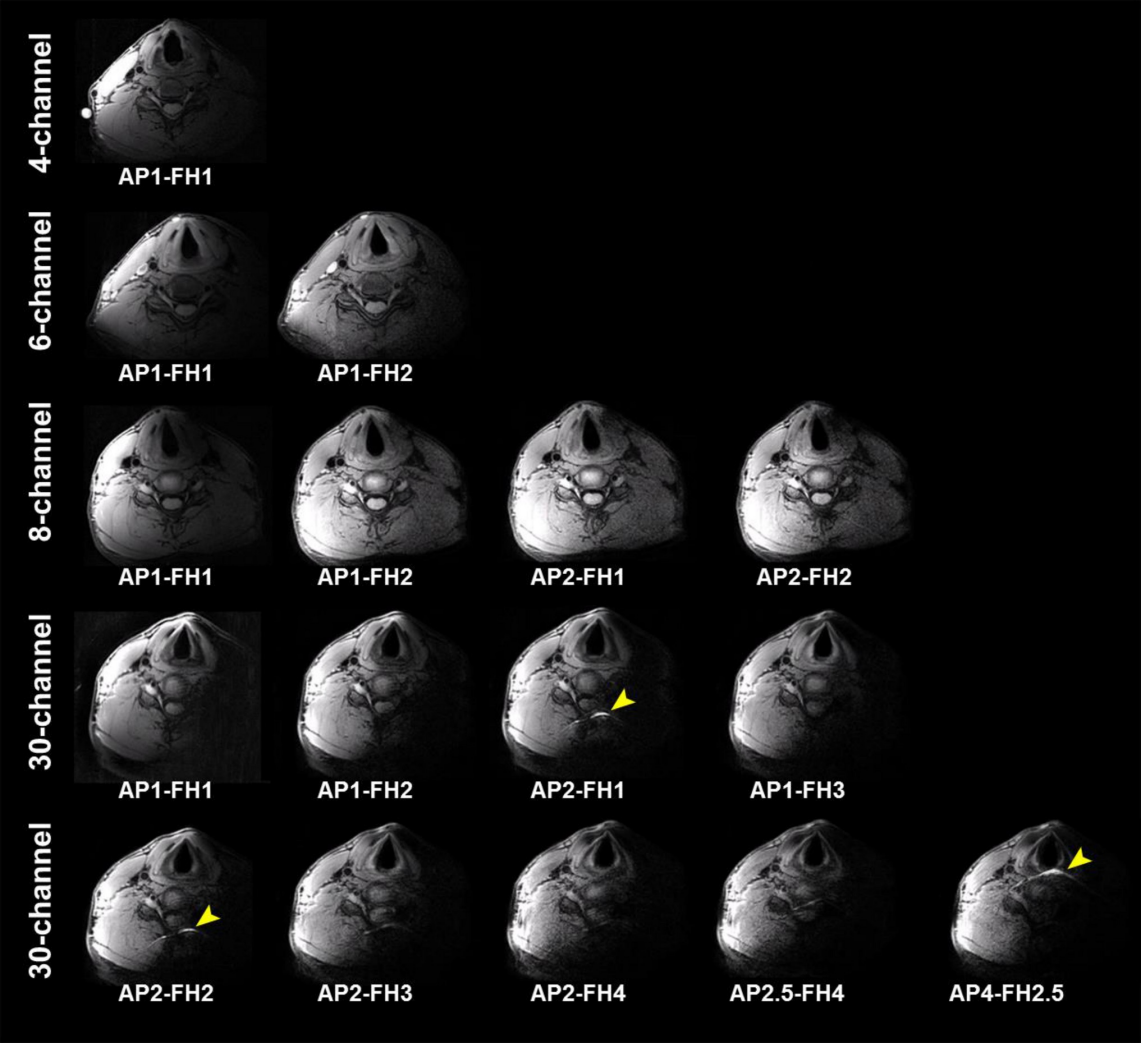
Representative transversal 3D‐MERGE images of the neck from one volunteer with all SENSE acceleration settings for the four coils. Minor SENSE fold-over artifacts for the 30-channel coil are indicated by yellow arrows.

**Fig 6:**
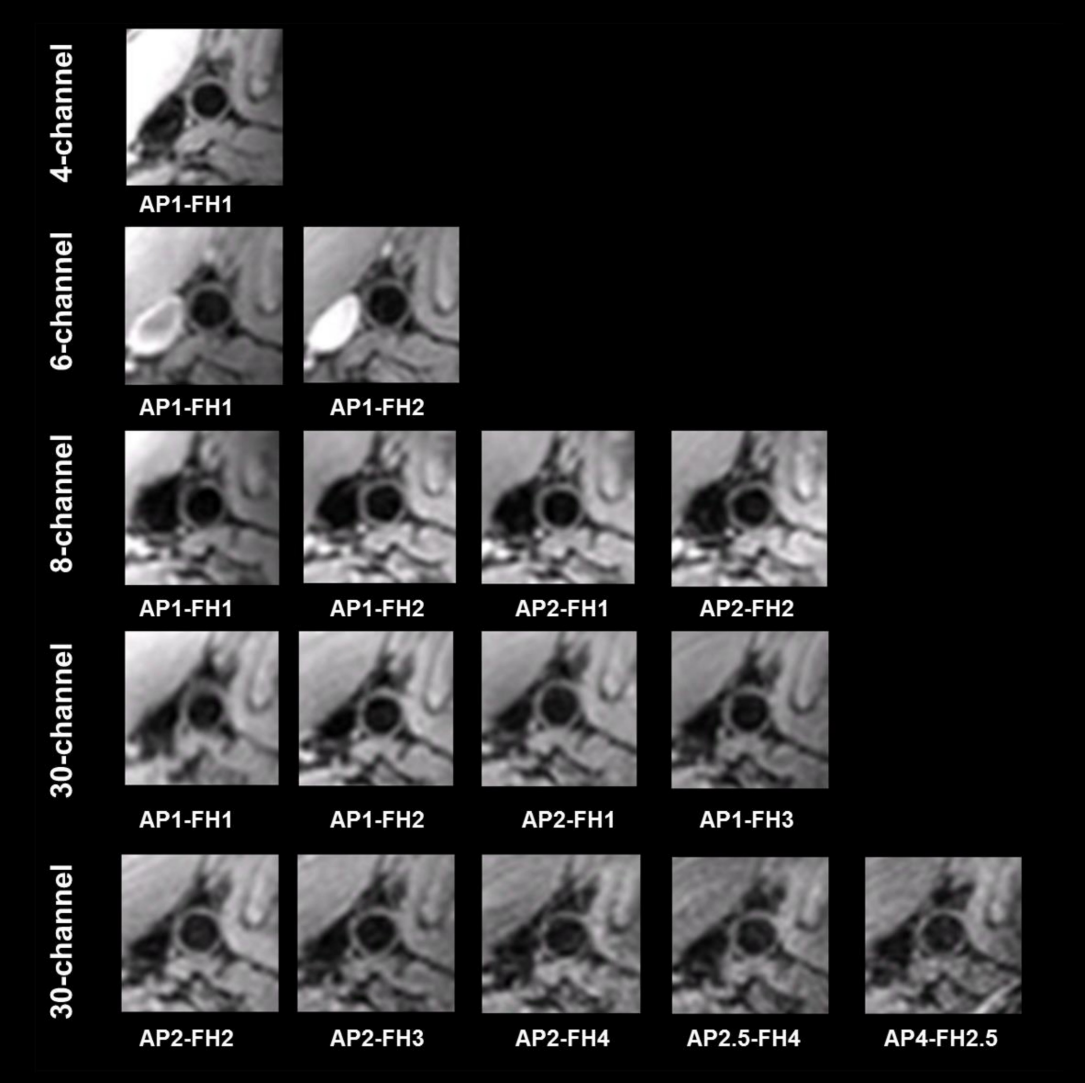
Magnified views of the carotid vessel from the images in Fig 5.

In Fig 5 representative images are shown of a transversal acquisition with all the proposed SENSE acceleration settings through the common carotid artery of another volunteer. For all the images, anatomical structures were clearly visible for the right side of the neck. Particularly, images from the 8-channel coil showed nearly full FOV coverage with SENSE factor up to 4. SNR decreased as the SENSE acceleration factor increased for all coils, which is more noticeable in deeper areas. No noticeable SENSE fold-over artifacts were present in the images from 4-, 6- and 8-channel coils. For the 30-channel coil, a minor fold-over artifact from subcutaneous fat located in the middle was visible when SENSE was applied in the AP direction. Images in the coronal view for the same volunteer are shown in the supplementary material (S1 Fig). All measurements were free of fold-over artifact in the FH direction. The transversal zoomed-in images of the common carotid are shown in Fig 6, in which artifact-free vessel wall could be clearly identified with moderate to high SNR for all the images. Note that in this volunteer, venous blood signal was not sufficiently suppressed for 6-channel coil measurements, which we believe is due the venous blood velocity being reduced below the venc threshold (3 cm/s), probably as result of tight attachment of the coil around the neck.

**Fig 7:**
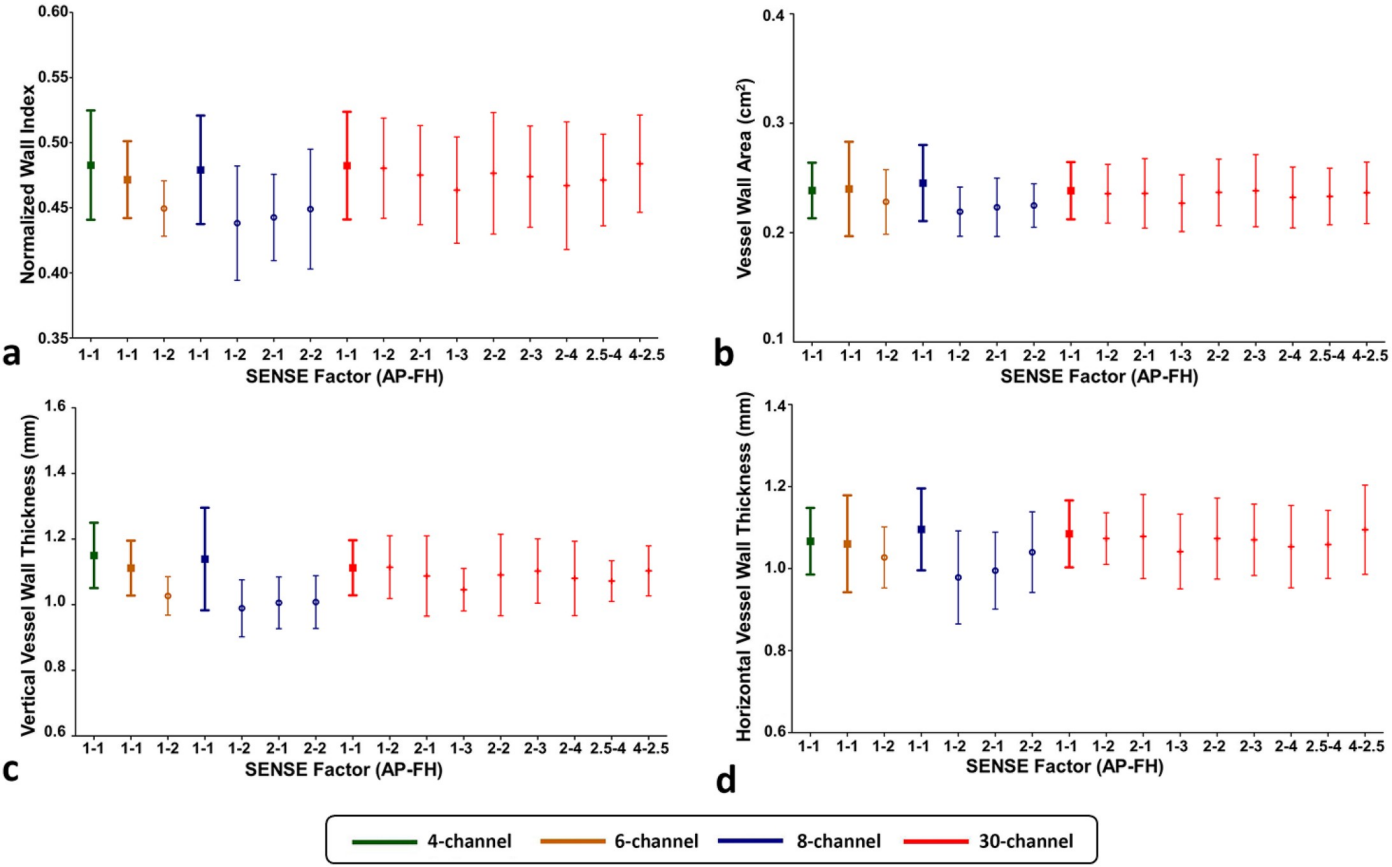
Bar plots of carotid vessel wall morphology parameters for all coils: (**a**) normalized wall index (NWI), (**b**) vessel wall area (A_vw_), (**c**) vertical vessel wall thickness (T_v_) and (**d**) horizontal vessel wall thickness (T_h_). For all the parameters, no significant differences were found for different coils or SENSE settings.

Mean and SD values of parameters describing vessel wall morphology including NWI, vessel wall area (A_vw_), vertical vessel wall thickness (T_v_) and horizontal vessel wall thickness (T_h_) are depicted in Fig 7. We found no significant effect of coil on all morphological parameters (*Fs* ≤ 0.50; *p* ≥ 0.70). In terms of the SENSE effect, for the 30-channel coil, we found no significant effect for all morphological parameters (*F*s ≤ 1.06; *p* ≥ 0.40). For the 6-channel coil, we found larger NWI (*p* = 0.04) and T_v_ (*p* = 0.001) for non-accelerated measurements compared to SENSE measurements (AP1-FH2). For the 8-channel coil, we found significant effects of SENSE on all vessel wall morphological parameters (*F*s > 5.30; *p*s < 0.01). Subsequent post-hoc tests showed significantly difference between the non-accelerated measurements and SENSE accelerated measurements. In detail, non-accelerated measurements had larger NWI (*p*s ≤ 0.03), A_vw_ (*p*s ≤ 0.05) and T_v_ (*p*s ≤ 0.02) compared to all SENSE measurements (AP1-FH2; AP2-FH1; AP2-FH2). Non-accelerated measurement also had larger T_h_ values (*p*s < 0.03) than AP1-FH2 and AP2-FH1 SENSE measurements. Other comparisons were not significant.

**Fig 8:**
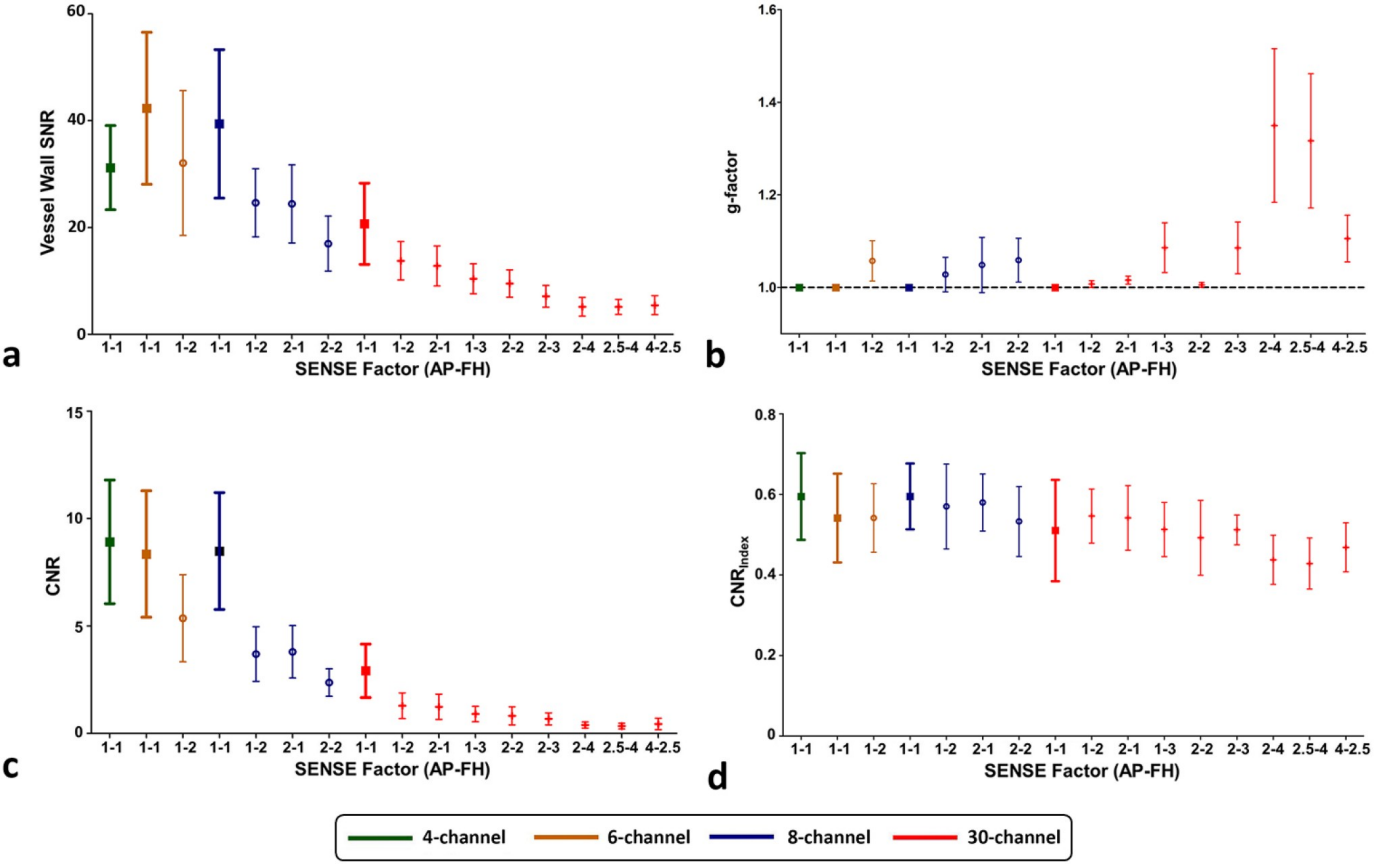
Bar plots of carotid image quality parameters for all coils: (**a**) vessel wall SNR, (**b**) g-factor, (**c**) vessel wall-lumen CNR and (**d**) vessel wall-lumen CNR_index_.

Fig 8 shows bar plots of image quality parameters including carotid vessel wall SNR, g-factor within vessel area, vessel wall-lumen CNR and CNR_Index_. Similar to Fig 7, parameter mean and SD values are plotted for different coils and SENSE settings. For the vessel wall SNR (Fig 8a), we found significant effect of coil (*F* = 20.77; *p* = 0.001). Following post-hoc tests showed this was a result of significantly lower SNR for the 30-channel coils compared to all other coils (*p*s ≤ 0.01) and significant lower SNR for the 4-channel coil compared to the 6-channel coil (*p* = 0.007). Moreover, we found significant SENSE effect for all coils (*F*s ≥ 26.71; *p*s ≤ 0.001) as SNR values were significantly lower for higher SENSE acceleration factors in most cases. However, several exceptions were found for the 30-channel coil: no significant differences were found for comparisons of AP1-FH3 versus AP2-FH1 (*p* = 0.06), AP2-FH2 versus AP1-FH3 (*p* = 1.00), AP2-FH3 versus AP2-FH2 (*p* = 0.06), AP2-FH4 versus AP2-FH3 (*p* = 0.27), AP4-FH2.5 versus AP2-FH3 (*p* = 0.66), AP2.5-FH4 versus AP2-FH4 (*p* = 1.000) and AP4-FH2.5 versus AP2-FH4 (*p* = 1.00).

Additional SNR loss beyond SENSE acceleration is described by the g-factor (Fig 8b). G-factor values for the 30-channel with SENSE settings of AP1-FH2, AP2-FH1 and AP2-FH2 were below 1.1 with SD smaller than 0.01, which were significantly lower than 6- and 8-channel coils under the same settings (*p*s ≤ 0.01). Moreover, the g-factor of the 30-channel coil remained low for SENSE factors of AP1-FH3 (1.1 ± 0.1), AP2-FH3 (1.1 ± 0.1) and AP4-FH2.5 (1.1 ± 0.1). The 30-channel g-factor increased for SENSE factor of AP2-FH4 (1.3 ± 0.2) and AP2.5-FH4 (1.4 ± 0.2), where the SENSE factor was larger than the number of coil elements in FH direction.

Fig 8c shows the CNR, i.e. the SNR difference between vessel wall and lumen for different coils and SENSE settings, which had similar patterns to the vessel wall SNR subplot as shown in Fig 8a. For CNR_Index_ (Fig 8d), significant effect of coil (*F* = 9.97; *p* = 0.01) was found due to significantly lower CNR_Index_ for the 30-channel coil compare to the 8-channel coil (*p* = 0.01). Additionally, significant SENSE effect was found for the 30-channel coil (*F* = 71.11; *p* = 0.001). Post-hoc tests showed SENSE settings of AP2-FH4 and AP2.5-FH4 had lower CNR_Index_ than AP1-FH2 and AP2-FH1 (*p*s ≤ 0.04). Other comparisons were not significant.

## Discussion

In this study, four carotid coils achieved similar results for vessel wall morphological parameters. The choice of different SENSE settings did not affect the vessel wall morphology parameters for the 30-channel coil. In contrast, the use of SENSE acceleration slightly decreased some parameter values for the 8-channel coil, which may be attributed to less motion interference for faster SENSE measurements. For all measurements and all volunteers, mean and SD values for NWI, A_wv_, T_v_ and T_h_ were 0.47 ± 0.04, 0.23 ± 0.03 cm^2^, 1.08 ± 0.10 mm and 1.04 ± 0.10 mm, respectively. Similar values were found in previous studies using comparable image resolutions [24, 27, 28]. However, these values were generally larger than those reported in a previous study using higher in-plane image resolution of 0.25 × 0.25 mm^2^ [29], as A_wv_ and vessel wall thickness decrease with increasing resolution [17, 29]. The resolution in this study was chosen to achieve large FOV coverage in the FH direction while maintaining a reasonable scan duration.

For SNR, g-factor and CNR performances, the four tested coils differed from each other due to differences in coil designs. Although these differences did not directly lead to different vessel wall morphology parameters in this study, the choice of coil may still be relevant in other carotid scanning protocols.

The 8-channel coil demonstrated high SNR performance with sufficient coverage in both FH and LR directions. The g-factor of the 8-channel coil remained below 1.25 for SENSE factor of 2 in both AP and FH directions. These properties promote its application in various carotid measurements, such as T_1_ weighted (T_1_w) imaging, T_2_ weighted (T_2_w) imaging, proton density weighed (PDw) imaging, and time of flight (TOF) imaging, as reported in previous studies [17, 18, 30].

The 6-channel coil showed great SNR performance similar to the 8-channel coil. Single-loop coil elements in the 6-channel coil were overlapped leading to smooth and uniform images with minimal signal intensity gap. This coil demonstrated big coverage in the FH direction as its three coil elements were aligned in the FH direction. However, the drawback of this design is that the degree of parallel imaging acceleration is fixed in the FH direction. When coronal or sagittal scanning is required, parallel imaging cannot be fully exploited for this coil.

The 4-channel coil had slightly worse SNR performance compared to the 6- and the 8-channel coils. Despite its coil elements were separated by 0.83 cm which cannot totally eliminate mutual inductance, this design had excellent electrical decoupling performance leading to low noise correlations between channels [31] (Fig 4). Additionally, this coil had smaller FH coverage compared to other coils as coil elements are aligned in the AP direction. The limited coil elements prevented the use of high SENSE factors but the small coil dimension could improve patient acceptance, ease of placement and could lead to less motion artifacts [19]. However, the small coverage along the carotid vessels may make it difficult to place the coil accurately at the region of interest such as the carotid bifurcation to obtain optimal SNR.

The 30-channel coil displayed inferior SNR penetration performance and exhibited obvious signal drop-offs between coil elements compared to the other coils. This may be caused by the small coil elements, which were arranged with large separation (1.47 cm) in the AP direction to facilitate optimal parallel imaging. A similar design was used for head coils previously [32, 33]. To confirm that the SNR inferiority could be mostly attributed to the coil design, an additional Q measurement [34] was done, which showed the resistive loss contributed only 7% to the total SNR penalty. By contrast, the outstanding parallel imaging performance of the 30-channel coil was illustrated by constant high quality 3D‐MERGE images with acceleration factors up to 10. These high parallel imaging factors are especially appealing for 3D imaging and high field carotid imaging where image SNR is inherently high. Using the same coil, we previously reported on 10-fold accelerated high spatiotemporal resolution 4D flow carotid imaging in only 7 min [35]. However, this high parallel imaging performance may be less attractive in 2D measurements for which the potential for SENSE acceleration and image SNR are limited.

This study has some limitations. First, all the four coils were only investigated unilaterally, because we did not have access to the left side of the 30-channel coil. However, the comparisons made in this study are still applicable, since there is no restriction to using the 30-channel coil bilaterally as previously demonstrated [36]. The bilateral setup could increase the image SNR of carotid vessels and enable parallel imaging in the LR direction. However, as the LR direction was the frequency encoding direction in this study, it was not feasible to apply SENSE in the LR direction. The possible SENSE settings in AP and FH directions were well explored by the unilateral setup in this study. Second, only one sequence (3D-MERGE) was evaluated on healthy volunteers in this study. Coil performances for other sequences and other interesting tissue types such as carotid plaque were not evaluated.

## Conclusion

In this study, we showed that all of the four dedicated carotid surface coils allowed local high SNR values > 20 for the chosen scanning sequence enabling high-resolution carotid artery imaging. The coils achieved similar carotid vessel wall morphology performance. Parallel imaging performance in different anatomical orientations depended on the specific coil element layout, which should be considered in study design. The high-density array 30-channel coil allowed parallel imaging acceleration factors up to 10 with only minimal penalties in g-factor noise. The coils with fewer elements (6 and 8) but bigger loop dimensions have excellent SNR performance, which is particularly attractive for 2D measurements and for SNR deprived quantitative mapping measurements.

## Supporting information

S1 Fig Representative coronal 3D‐MERGE images of the neck for the same volunteer shown in Fig 5, with all SENSE acceleration settings for the four coils. All measurements are free of SENSE fold-over artifacts in the feet-head (FH) direction.
(DOC)
