# Supplementary material for: Comparison of four MR carotid surface coils at 3T
Source: PLoS One. 2019 Mar 4;14(3):e0213107. doi: 10.1371/journal.pone.0213107 (PMC6398924; doi:10.1371/journal.pone.0213107)
Supplement: S1 Fig — All measurements are free of SENSE fold-over artifacts in the feet-head (FH) direction. (DOC) [file pone.0213107.s001.doc]

**Supplementary material for “Comparison of Four MR Carotid Surface Coils at 3T”**

Qinwei Zhang, Bram F. Coolen, Sandra van den Berg, Gyula Kotek, Debra S. Rivera, Dennis. W. J. Klomp, Gustav. J. Strijkers, Aart. J. Nederveen


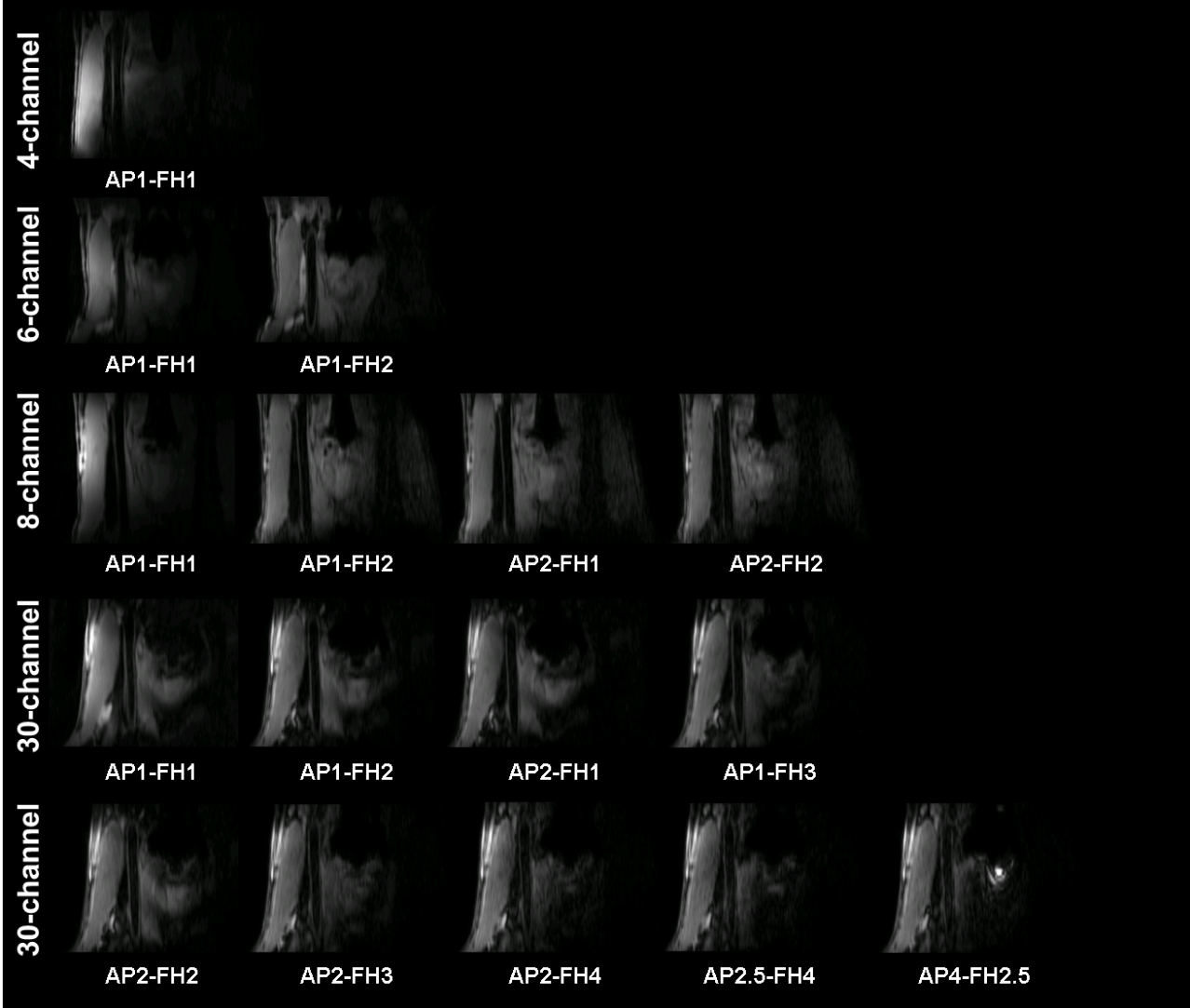


**S1 Fig**: Representative coronal 3D‐MERGE images of the neck for the same volunteer shown in **Fig 5,** with all SENSE acceleration settings for the four coils. All measurements are free of SENSE fold-over artifacts in the feet-head (FH) direction.
